# Temporal variations in ambient air quality indicators in Shanghai municipality, China

**DOI:** 10.1038/s41598-020-68201-0

**Published:** 2020-07-09

**Authors:** Yuanyuan Chen, Yang Bai, Hongtao Liu, Juha M. Alatalo, Bo Jiang

**Affiliations:** 1 CAS Key Laboratory of Aquatic Botany and Watershed Ecology, Wuhan Botanical Garden, Chinese Academy of Sciences, Wuhan, 430074 China; 2 Changjiang Water Resources Protection Institute, Wuhan, 430051 China; 3 Center for Integrative Conservation, Xishuangbanna Tropical Botanical Garden, Chinese Academy of Sciences, Xishuangbanna, 666303 China; 4 University of Chinese Academy of Sciences, Beijing, 100049 China; 5 Department of Biological and Environmental Science, College of Arts and Sciences, Qatar University, P.O. Box 2713, Doha, Qatar; 6 Environmental Science Center, Qatar University, P.O.Box: 2713, Doha, Qatar

**Keywords:** Environmental impact, Urban ecology

## Abstract

Official data on daily PM_2.5_, PM_10_, SO_2_, NO_2_, CO, and maximum 8-h average O_3_ (O_3__8h) concentrations from January 2015 to December 2018 were evaluated and air pollution status and dynamics in Shanghai municipality were examined. Factors affecting air quality, including meteorological factors and socio-economic indicators, were analyzed. The main findings were that: (1) Overall air quality status in Shanghai municipality has improved and number of days meeting ‘Chinese ambient air quality standards’ (CAAQS) Grade II has increased. (2) The most frequent major pollutant in Shanghai municipality is O_3_ (which exceeded the standard on 110 days in 2015, 84 days in 2016, 126 days in 2017, 113 days in 2018), followed by PM_2.5_ (120days in 2015, 104 days in 2016, 67 days in 2017, 61 days in 2018) and NO_2_ (50 days in 2015, 67 days in 2016, 79 days in 2017, 63 days in 2018). (3) PM_2.5_ pollution in winter and O_3_ pollution in summer are the main air quality challenges in Shanghai municipality. (4) Statistical analysis suggested that PM_2.5_, PM_10_, SO_2_ and NO_2_ concentrations were significantly negatively associated with precipitation (Prec) and atmosphere temperature (T) (p < 0.05), while the O_3_ concentration was significantly positively associated with Prec and T (p < 0.05). Lower accumulation of PM, SO_2_, NO_2_, and CO and more serious O_3_ pollution were revealed during months with higher temperature and more precipitation in Shanghai. The correlation between the socio-economic factors and the air pollutants suggest that further rigorous measures are needed to control PM_2.5_ and that further studies are needed to identify O_3_ formation mechanisms and control strategies. The results provide scientific insights into meteorological factors and socio-economic indicators influencing air pollution in Shanghai.

## Introduction

China’s reforms and opening-up policies since 1970s have contributed to rapid economic growth, industrialization, and urbanization^1,2^, as evidenced by increased gross domestic product (GDP), urban population, and energy consumption^1,3,4^. However, this has resulted in high levels of environmental degradation^1,5,6^ and associated health effects^2,6^. Air pollution in China is mainly caused by coal combustion, motor vehicles, industrial dust, chemical conversion in the atmosphere in urban centers, and unfavorable meteorological conditions, all of which are linked to rapid socioeconomic development^1,3,7,8^. With an increasing number of Chinese cities suffering from serious air pollution problems in recent decades^1,2,9^, air pollution has become one of the top environmental concerns in China^1,6,9–13^. Serious air pollution hinders economic development^14^ and deteriorates people’s quality of life, with increasing reports of negative health risks^6,15^. Many epidemiological studies have shown that air pollution has strong associations with impaired human health^16^ and mortality^14,16,17^. A recent study found that a 10 μg m^−3^ increase in particulate matter (PM_10_) reduced life expectancy in China by 0.64 years^18^. Other studies in China have estimated that a 10 μg m^−3^ increase in PM_10_ led to a 0.44% increase in daily number of deaths^19^, that PM_2.5_ accounted for 15.5% (1.7 million) of all-cause deaths in China in 2015^20^, and that 2.19 million (2013), 1.94 million (2014), and 1.65 million (2015) premature deaths could be attributed to long-term exposure to PM_2.5_^21^. However, a recent study estimated that the number of premature deaths in China attributable to PM_2.5_ has decreased by 12.6%, from 1.20 million in 2013 to 1.05 million in 2017^22^.

With the growing need for improving air quality across cities, municipalities, and provinces in China, a series of laws, regulations, standards and control measures have been formulated and promulgated^1,2,4,8,23^. The ‘Air Pollution Prevention Action Plan’ was enacted on September 10, 2013, and the most stringent environmental protection law to date was implemented on January 1, 2015^8^. Significant measures have also been taken to mitigate the adverse effects of air pollution^24^. Air quality monitoring systems have been established in more than 330 cities^16^ and at 1,300 national air quality monitoring sites^24^. Daily data on air quality index (AQI) and air quality indicators are released publicly on local government websites, providing an important foundation for air quality research and policy. In the past three decades, knowledge on air pollution has improved considerably with the growing number of publications on air pollution in megacities^2,4,8,14,16,22,24,25^. Many studies have reported spatio-temporal variations in particulate matter (PM_2.5_ and PM_10_) and gaseous (SO_2_, NO_2_, CO, and O_3_) pollutants in Chinese cities^4,8,16,24,26^, and associated health and socioeconomic costs^3,6,14,21,22,27–29^. Between 2013 and 2018, China’s rigorous air pollution control greatly reduced the annual mean level of PM_2.5_ in the atmosphere of 74 large cities^30^.

Shanghai is an important political, economic, and cultural center of China. With the acceleration of urbanization and industrial processes, Shanghai’s environmental problems have become increasingly prominent, with air quality being one of the most serious issues. As a pioneer city in construction of ecological civilization, Shanghai’s air quality has received much attention. In this study, official data on daily concentrations of PM_2.5_, PM_10_, SO_2_, NO_2_, CO, and maximum 8-h average concentration of O_3_ (O_3__8h) in the air in Shanghai municipality from January 2015 to December 2018 were used to examine air pollution status and dynamics in the municipality. The following aspects are addressed in this paper: (1) Temporal variations in average daily concentrations of PM_2.5_, PM_10_, SO_2_, NO_2_, CO, and O_3__8h in the air in Shanghai municipality during 2015–2018; (2) annual and seasonal variations in major pollutants and number of days when concentrations exceeded the air quality standard; and (3) the main meteorological factors and socio-economic indicators affecting air pollution in Shanghai. The results were used to identify air quality management gaps in the municipality.

## Results and discussion

### Overview of air pollutants in Shanghai during 2015–2018

**Figure 1:**
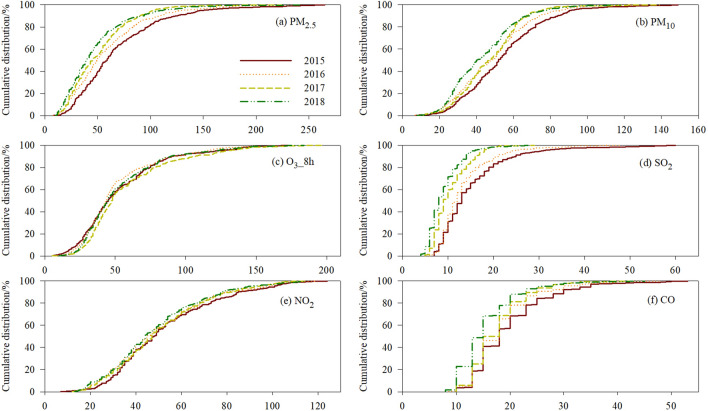
(**a**–**f**) Cumulative distribution of daily average mean concentrations of air pollutants in Shanghai municipality.

The average mass concentrations of the target pollutants during 2015–2018 were analyzed. We used the cumulative distribution of daily average values of PM_2.5_, PM_10_, NO_2_, SO_2_, CO, and O_3__8h to determine the number of days during which Shanghai municipality was exposed to air pollution (Fig. 1)^24^. For at least some half-days in 2015 (2016, 2017, 2018), Shanghai municipality was exposed to average values higher than 59 (50, 45, 40) μg m^−3^ for PM_2.5_, 52 (48, 47, 40) μg m^−3^ for PM_10_, 45 (43, 47, 44) μg m^−3^ for O_3__8h, 48 (45, 47, 44) μg m^−3^ for NO_2_, 13 (12, 9, 8) μg m^−3^ for SO_2_, and 18 (18, 18, 15) mg m^−3^ for CO. This indicates a decrease in the number of days per year in which Shanghai residents were exposed to high concentrations of PM_2.5_, PM_10_, NO_2_, SO_2_, and CO.

### Temporal variations in air pollutants

**Figure 2:**
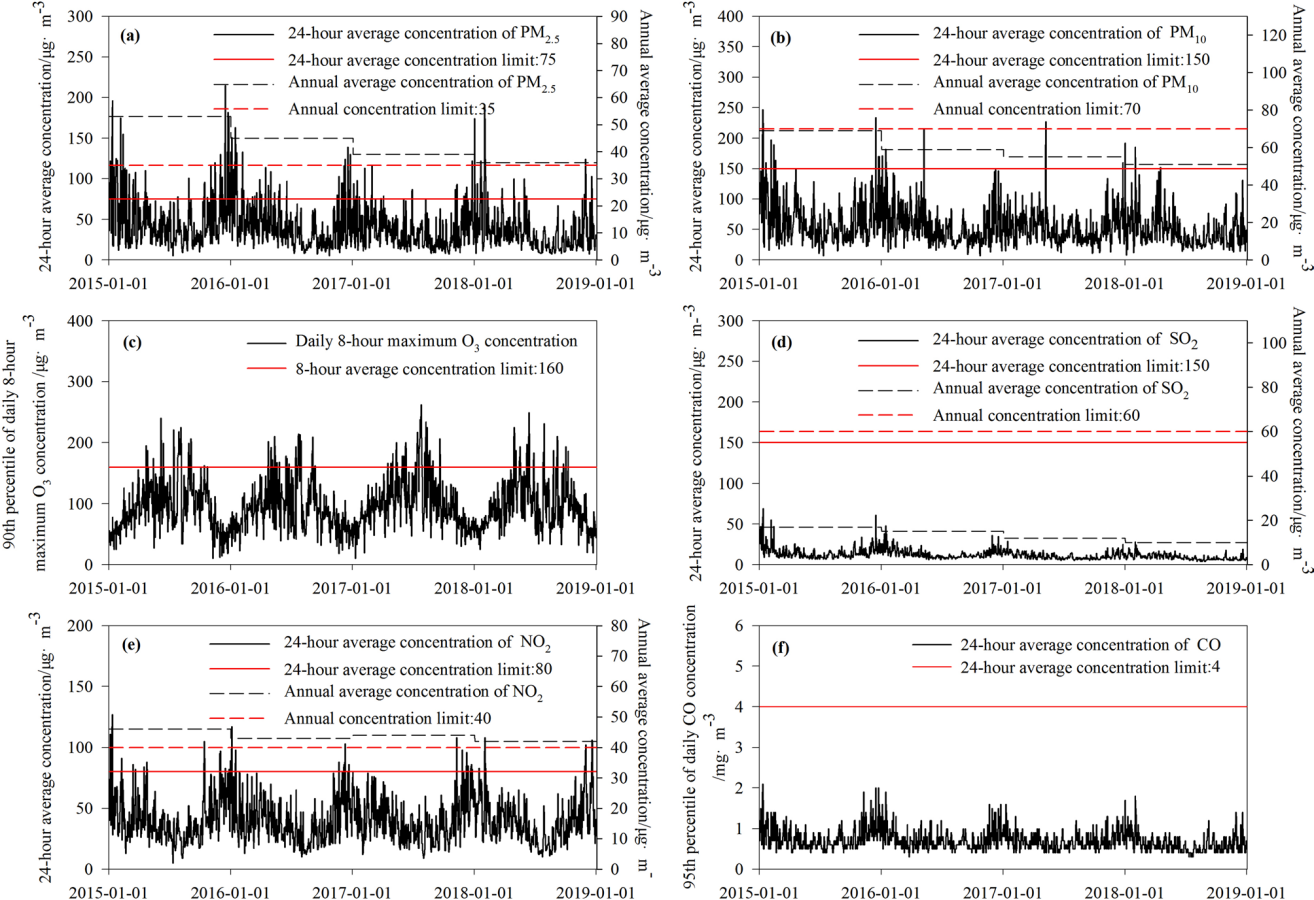
Temporal variations in 24-h average concentrations and annual mean concentrations of air pollutants in Shanghai municipality, 2015–2018.

Following implementation of the six-round, 3-year environmental protection action plan, ambient air quality in Shanghai municipality has improved slightly. In 2018, the average annual concentration of SO_2_ and PM_10_ in Shanghai municipality was 10 μg m^−3^ and 51 μg m^−3^ respectively, the 90th percentile of O_3__8h concentration was 160 μg m^−3^, and daily CO concentration was within the range 0.4–2.0 mg m^−3^. All these concentrations met the national Level I or Level II for annual mean ambient air quality. However, the average annual concentration of NO_2_ and PM_2.5_ in the city in 2018 was 42 μg m^−3^ and 36 μg m^−3^, respectively, which did not meet the Level II annual mean level air quality standard. Moreover, monitoring data for the past 4 years show that the annual mean concentrations of NO_2_ and PM_2.5_ in Shanghai are generally declining, but they still exceed the national Level II air quality standards. The daily maximum 8-h average, 24-h average, and annual mean concentrations of six air pollutants in Shanghai municipality during 2015–2018 are summarized in Fig. 2. Compared with 2015, the average concentration in 2018 decreased by 32.08%, 26.09%, 0.62%, 41.18%, 8.70%, and 22.09% for PM_2.5_, PM_10_, O_3__8h, SO_2_, NO_2_, and CO, respectively. The large decrease in SO_2_ in the air Shanghai municipality was consistent with the overall trend in annual mean concentration of SO_2_ in China^8^. This indicates effective control of combustion emissions and implementation of desulfurization systems^8,31^. Our results also indicated that more than 70% of the total mass of PM_10_ was composed of PM_2.5_, which is close to the ratio reported in previous studies^8,24^. The decreases in CO and NO_2_ concentrations were mainly attributable to effective regulation of coal combustion emissions and traffic-related emissions^8,31–33^. The reductions amplitudes were lower for CO and NO_2_ compared with PM_2.5_, PM_10_, and SO_2_, which may be related to the rapid increase in vehicles in Chinese cities^8^. No clear decrease was observed for the 90th percentile of O_3__8h concentration in this study. Air pollution has gradually changed from the conventional coal combustion type to mixed coal combustion/motor vehicle emission type^3^, reflecting the rapid increase in the number of motor vehicles in Shanghai municipality^34^. This poses enormous challenges for air pollution control and environmental management.

### Major pollutants and non-attainment days

**Figure 3:**
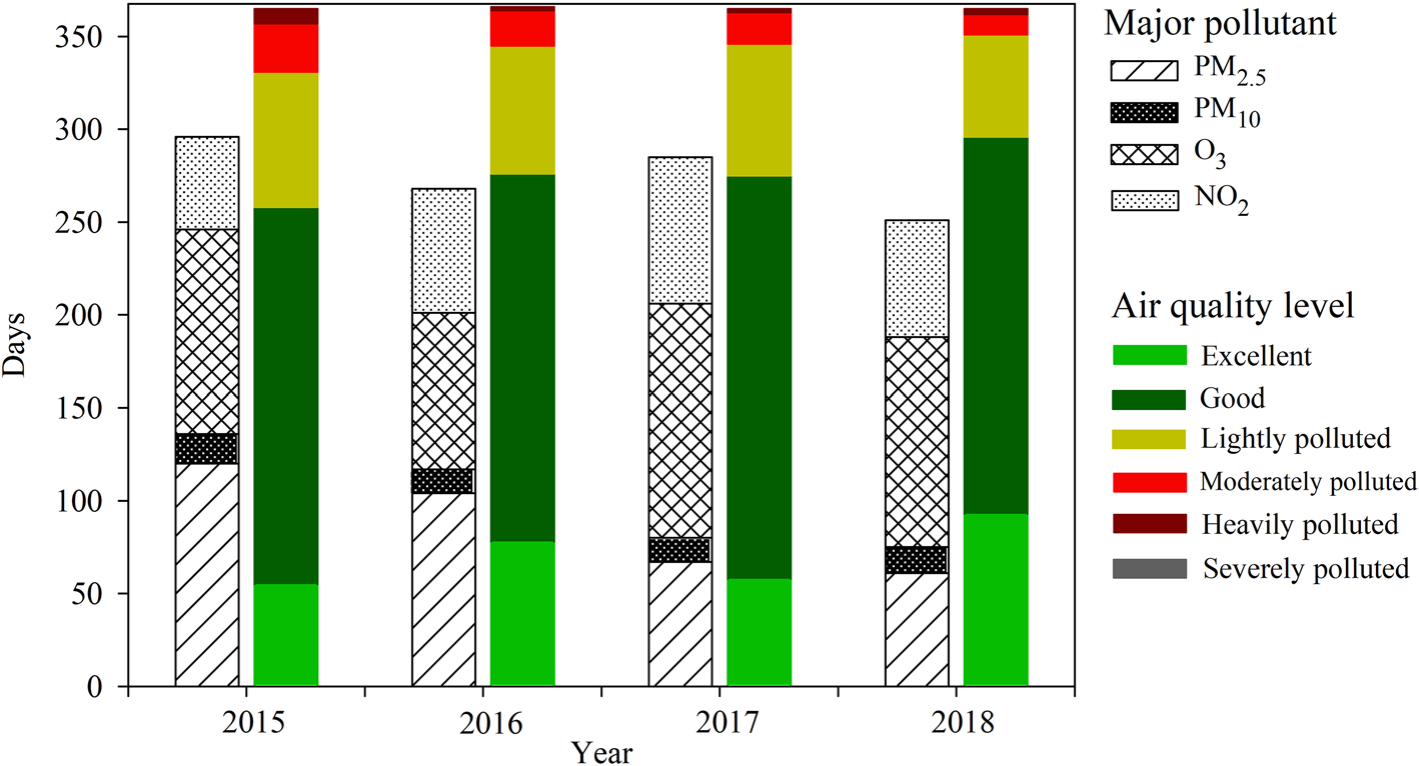
Number of days per year on which each pollutant was designated a “major pollutant” (different shapes) and air quality level (different colors) in Shanghai municipality.

The number of days meeting the mean concentration limits of ‘Chinese ambient air quality standards’ (CAAQS) in Shanghai municipality during 2015–2018 was examined (Fig. 3). In 2015 (2016, 2017, 2018), 18.6% (27.5%, 33.6%, 41.5%), 77.9% (85.3%, 92.6%, 91.5%), 35.8% (40.1%, 35.2%, 41.0%), 99.5% (100%, 100%,100%), 99.7 (100%, 100%, 100%), and 58.4% (67.2%, 57.0%, 60.8%) of days met the concentration limit in CAAQS Grade II for 24-h average PM_2.5_, PM_10_, NO_2_, SO_2_, CO, and maximum 8-h average O_3_. Compared with 2015, the number of days in 2018 that met the level in CAAQS Grade II increased by 124.3%, 17.5%, 4.1%, 14.5%, 0.5%, and 0.3% for PM_2.5_, PM_10_, O_3__8h, SO_2_, NO_2_, and CO, respectively. The number of days with excellent air quality increased from 55 in 2015 to 93 in 2018, while the number of days with ‘good’ air quality remained consistent at 203 days between 2015 and 2018.

The most frequent “major pollutant” in Shanghai municipality was O_3_, followed by PM_2.5_ and then NO_2_ and PM_10_. In comparison, SO_2_ and CO were the “major pollutant” considerably less frequently. The number of days on which PM_2.5,_ O_3_, NO_2_, and PM_10_ was designated the “major pollutant” was 120 (104, 67, 61), 110 (84, 126, 113), 50 (67, 79, 63) and 16 (13, 13, 14) in 2015 (2016, 2017, 2018), respectively. The low incidence of SO_2_ as a “major pollutant” again indicated effective control of coal combustion and implementation of desulphurization systems^8,31^. Compared with 2015, the incidence of O_3_ as a major pollutant in Shanghai increased to reach its highest value in 2017. This is consistent with the 90th percentile of O_3__8h concentration, which also peaked in 2017. Previous studies have suggested that O_3_ is a complex secondary pollutant related to solar radiation, NO_x_, volatile organic compounds (VOC), and vertical transport in the boundary layer^8^, factors that are difficult to control effectively^35,36^. While the number of polluted days with PM_2.5_ concentrations over 75 μg m^−3^ decreased from 2015 to 2018, the complex mixture of PM_2.5_ and O_3_ in the air is still a challenge to continuous improvement of air quality in Shanghai municipality^8,24^.

**Figure 4:**
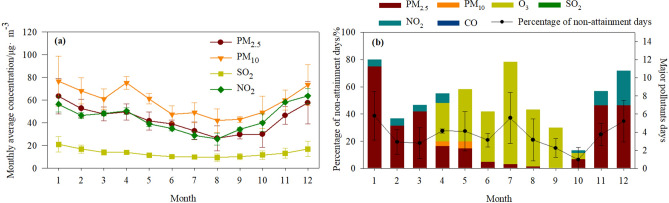
(**a**) Average concentration of the pollutants PM_2.5_, PM_10_, SO_2_, and NO_2_ and (**b**) percentage of non-attainment days and major pollutant on polluted days in each month during 2015–2018.

There were seasonal variations in the concentrations of each pollutant (Fig. 4a), and thus the days on which the air quality standard was exceeded (non-attainment days) were not equally distributed throughout the year (Fig. 4b), which is consistent with findings in previous studies^24,37^. November, December, January, February, and March were the dominant months with non-attainment days for PM_2.5_ in Shanghai municipality, while April, May, June, July, August, and September were the dominant months with non-attainment days for O_3__8h. Overall, winter months had the largest number of polluted days and highest mean concentration of PM_2.5_, followed by spring, autumn, and summer, which is consistent with previous findings^16^. This trend has been mainly attributed to coal-fired heating of buildings^16,38–40^. Summertime O_3_ pollution in Shanghai was much more severe than in the other seasons (Fig. 4b), and the probability of O_3__8h exceeding the CAAQS Grade II value was highest in July (11.25 ± 5.85 day), followed by August (6.25 ± 4.65 day), May (5.75 ± 3.2 day), and June (5.5 ± 1.29 day). This is consistent with findings in previous studies that summer is the O_3_ episode season in Chinese megacity clusters^41,42^. Polluted days with NO_2_ > 80 μg m^−3^ were mainly observed during winter and spring. The low probability of SO_2_ exceeding the CAAQS Grade II value reflected the stringent SO_2_ emission regulations in Shanghai municipality^31^.

### Correlations between air pollutants

Different air pollutants were significantly correlated (*p* < 0.01) with each other, except for SO_2_ and O_3_ (Table 1). There were significant positive correlations between PM_2.5_, PM_10_, CO, SO_2_, and NO_2_, suggesting that these pollutants originated from the same sources (e.g., vehicle and coal emissions) or were impacted by the same drivers^24^. Therefore controlling traffic and coal combustion emissions might be a way of simultaneously decreasing the concentrations of these pollutants. O_3_ was significantly positively correlated with PM, and negatively correlated with NO_2_ and CO (*p* < 0.01). The correlation coefficients were weaker, however, which can mainly be attributed to the complex, nonlinear, and temperature-dependent chemistry of O_3_ concentration^20,43^. This indicates difficulty in controlling O_3_ concentration and merits further investigations on O_3_ formation and control strategies in Shanghai municipality.

**Table 1:** Correlations between pollutants based on daily data for Shanghai during 2015–2018 (**p < 0.01; *p < 0.05).

	PM_10_	O_3_	SO_2_	NO_2_	CO
PM_2.5_	0.879**	0.093**	0.708**	0.693**	0.817**
PM_10_		0.172**	0.739**	0.632**	0.686**
O_3_			− 0.026	− 0.206**	− 0.128**
SO_2_				0.602**	0.633**
NO_2_					0.706**

### Correlations between air pollutants and meteorological factors

Correlations between the six main pollutants and meteorological factors are shown in Table 2. The results suggested that temperature (*T*) significantly impacted accumulation of all six pollutants in Shanghai municipality, while precipitation (*Prec*) and relative air humidity (*RH*) may have affected accumulation of some pollutants. Of all the meteorological factors that significantly impacted pollutant concentrations, the correlations between meteorological factors and PM_2.5_, PM_10_, CO, SO_2_, and NO_2_ were negative, while the correlations between meteorological factors and O_3_ were positive.

**Table 2:** Correlations between air pollutants and meteorological factors based on the monthly data for Shanghai during 2015–2018.

	*W*	*T*	*RH*	PM_2.5_	PM_10_	O_3_	SO_2_	NO_2_	CO
*Prec*	− 0.093	0.532**	0.765**	− 0.353*	− 0.435**	0.342*	− 0.459**	− 0.429**	− 0.289*
*W*		− 0.205	− 0.222	− 0.056	0.033	− 0.072	0.125	− 0.154	− 0.212
*T*			0.416**	− 0.77**	− 0.674**	0.735**	− 0.703**	− 0.839**	− 0.67**
*RH*			1	− 0.252	− 0.472**	− 0.015	− 0.403**	− 0.293*	− 0.185

*Prec*: precipitation; *W*: wind speed in two minutes; *T*: temperature; *RH*: relative air humidity.

**p < 0.01; *p < 0.05.

The concentrations of PM_2.5_, PM_10_, SO_2_, NO_2_, and CO displayed a significantly negative relationship with *Prec* (*p* < 0.05 or *p* < 0.01), suggesting that the wet deposition could mitigate air pollution by the scavenge and wash-out process^16,44,45^. Relative humidity was strongly positively correlated with *Prec*, leading consistently to significantly negative correlations between PM_10_, SO_2_ and NO_2_ and *RH*. The consistency in correlations between the pollutants and *T*, and that between the pollutants and *Prec*, was partly explained by the significantly positive correlation between *Prec* and *T*. This also explains why the average concentration of the pollutants PM_2.5_, PM_10_, SO_2_, and NO_2_ during June–September was lower than in other months^46,47^. Wind speed (*W*) did not show any marked relationship with the air pollutants studied, indicating that *W* did not enhance air ventilation and turbulence and thus improve air quality.

### Correlations between air pollutants and socio-economic indicators

**Figure 5:**
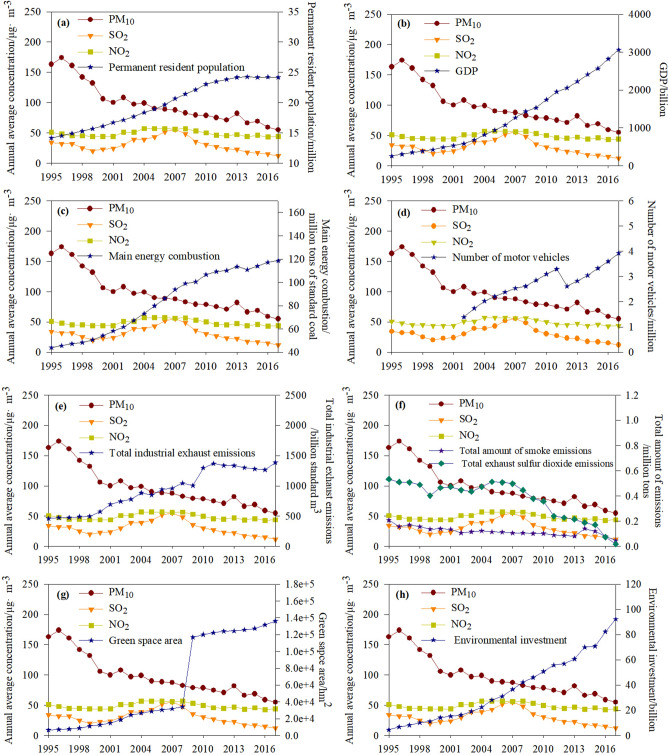
Annual change in average concentrations of three pollutants (PM_10_, SO_2_, NO_2_) relative to (**a**) permanent resident population, (**b**) gross domestic product (GDP), (**c**) energy combustion, (**d**) number of motor vehicles, (**e**) total industrial exhaust emissions, (**f**) total amount of smoke emissions and exhaust sulfur dioxide emissions, (**g**) green space area, and (**h**) environmental investment in Shanghai during 1995–2017.

Shanghai is undergoing strong socioeconomic development, with the permanent resident population (PRP) increasing from 14.14 million in 1995 to 24.18 million in 2017, and the GDP of Shanghai municipality increasing from 251.8 billion RMB in 1995 to 3,063.2 billion RMB in 2017^34^ (Fig. 5). In the same period, Shanghai municipality continuously increased its environmental protection and construction efforts, with rolling implementation of the six-round, 3-year environmental protection action plan. Green space area (GE) has increased, from 6,561 hm^2^ in 1995 to 136,327 hm^2^ in 2017, environmental investment (EI) has also increased, from 4.65 billion RMB in 1995 to 92.35 billion RMB in 2017, and total amount of smoke emissions (SE) and total exhaust sulfur dioxide emissions (SDE) has decreased from 207.8 thousand tons and 534.1 thousand tons, respectively, in 1995 to 47 thousand tons and 18.5 thousand tons, respectively, in 2017^34^ (Fig. 5). However, energy consumption (EC) has increased, from 4,392.48 × 10^4^ tons of standard coal in 1995 to 11,858.96 × 10^4^ tons of standard coal in 2017, the number of motor vehicles (MV) has increased, from 1.39 million in 2002 to 3.92 million in 2017^34^ (Fig. 5), and the volume of total industrial exhaust emissions (IEE) has increased, from 4,625 billion standard m^3^ in 1995 to 13,867 billion standard m^3^ in 2017^34^ (Fig. 5). Although ambient air quality in Shanghai municipality has improved slightly in recent decades as a result of its environmental regulations (Fig. 5), Shanghai is still one of the cities with the highest levels of air pollutants worldwide^48^.

The correlations between GS, IEE, SE, SDE, PRP, GDP, EC, MV, EI, and air concentrations of PM_10_, SO_2_ and NO_2_ are shown in Table 3. Although there have been large increases in PRP, GDP, EC, MV, and IEE in Shanghai in recent years, the increase in EI and the decrease in SE and SDE have compensated for the negative effects of the other factors, leading to positive effects in decreasing the concentrations of PM_10_, SO_2_, and NO_2_. The results revealed that investments in environmental protection and pollution control strategies were the main factors affecting accumulation of PM_10_, SO_2_, and NO_2_, indicating that such strategies are effective in reducing air pollution. The control in SE and SDE, and increase in EI and GS may be masking the increase in EC, MV, and IEE, leading to significant decrease in PM_10_, and slight decrease in NO_2_ and SO_2_. The increased vehicle emissions and main energy would also help explain the relative stability NO_2_ and SO_2_ levels. As a pioneering city in the construction of ecological civilization, Shanghai has implemented several master plans to optimize GS in integration with an environmental sustainability agenda^49^. The implementation of ecological redline policy in Shanghai municipality could guarantee that GS be increased systematically or stabilized at this level^50^ toward increasing the air quality. However, due to the lack in more detailed emission data per activity sector for all the pollutants, it is difficult to provide more concrete and quantitative evidence of the reasons that are driving the changes in the air quality, and explain if changes in air quality are really happening or if industrial sources are just getting better at not emitting the pollutants being monitored. Further studies are needed to reveal the percentage contribution of emission sources and atmospheric processes to the emissions of the pollutants.

**Table 3:** Correlations between pollutants and socio-economic indicators based on yearly data for the period 1995–2017.

	PM_10_	SO_2_	NO_2_
GS	− 0.984**	− 0.410	− 0.153
IEE	− 0.940**	− 0.328	− 0.080
SE	0.842**	0.144	− 0.051
SDE	0.699**	0.707**	0.491*
PRP	− 0.979**	− 0.401	− 0.159
GDP	− 0.837**	− 0.428*	− 0.153
EC	− 0.901**	− 0.192	− 0.145
MV	− 0.942**	− 0.602*	− 0.705**
EI	− 0.849**	− 0.417*	− 0.153

GS: green space area; IEE: total industrial exhaust emissions; SE: total amount of smoke emissions; SDE: total amount of exhaust sulfur dioxide emissions; PRP: permanent resident population; GDP: gross domestic product; EC: energy combustion; MV: number of motor vehicles; EI: environmental investment.

**p < 0.01; *p < 0.05.

## Conclusions

This study analyzed temporal variations in the concentrations of air pollutants (PM_2.5_, PM_10_, O_3_, SO_2_, NO_2_, and CO), the major pollutant on polluted days, and the number of non-attainment days in Shanghai municipality from January 2015 to December 2018. Based on 4-year data from the Shanghai Environmental Monitoring Center, the overall status of air quality in Shanghai has improved. The number of days that met CAAQS Grade II standards increased from 258 in 2015 to 296 in 2018.

We found that SO_2_ was rarely the “major pollutant”, indicating effective control of coal combustion and implementation of desulphurization system in Shanghai municipality. However, PM_2.5_ pollution in wintertime and O_3_ pollution in summertime are still major challenges to air quality improvement in Shanghai municipality. Our findings suggest that the most frequent major pollutant in Shanghai municipality is O_3_ (110 days in 2015, 84 days in 2016, 126 days in 2017, 113 days in 2018), followed by PM_2.5_ (120 days in 2015, 104 days in 2016, 67 days in 2017, 61 days in 2018) and NO_2_ (50 days in 2015, 67 days in 2016, 79 days in 2017, 63 days in 2018). O_3_ is a complex secondary pollutant that is difficult to control effectively. The non-clear decrease in O_3__8h concentration from 2015 to 2018 and a peak in O_3__8h concentration in 2017 indicate a need for further studies on O_3_ formation and control strategies.

Statistical analysis suggested that different air pollutants were significantly correlated with each other, apart from SO_2_ and O_3_. Significantly positive correlations between PM_2.5_, PM_10_, CO, SO_2_, and NO_2_ were observed, suggesting that these pollutants may have originated from the same sources (e.g., vehicle and coal combustion emissions) or were impacted by the same drivers. The correlation results suggested that temperature (*T*) significantly impacted accumulation of all six pollutants in Shanghai municipality, while precipitation (*Prec*) and relative air humidity (*RH*) affected accumulation of some pollutants. Lower accumulation of PM, SO_2_, NO_2_, CO and more serious O_3_ pollution in Shanghai were revealed in months with higher temperature and more precipitation. The correlation between the socio-economic factors and the air pollutants suggest that further rigorous measures are needed to control air pollution in the city. Investments in environmental protection and pollution control strategies were the main factors reducing accumulation of PM_10_, SO_2_, and NO_2_, indicating that these strategies are effective in reducing air pollution. Overall, this study provided scientific insights into impacts of meteorological factors and socio-economic indicators on air pollution in Shanghai.

## Methods

The most recent CAAQS were published in 2012^8,51^, when PM_2.5_ and O_3__8h were added for the first time^24^. These latest CAAQS set annual, 24-h average, and 1-h average concentration limits for SO_2_ and NO_2_, annual and 24-h average concentration limits for PM_2.5_ and PM_10_, 24-h average and 1-h average concentration limits for CO, and maximum 8-h average and 1-h average concentration limits for O_3_. In the same year, a ‘Technical Regulation on Ambient Air Quality Index (on trial)’ (HJ 633–2012) released by the Chinese Ministry of Environmental Protection (MEP)^52^ replaced air pollution index (API) with AQI and divided air quality into six classes: 0–50 (Level I, excellent), 51–100 (Level II, good), 101–150 (Level III, lightly polluted), 151–200 (Level IV, moderately polluted), 201–00 (Level V, heavily polluted), and above 300 (Level VI, severely polluted)^8,28^. Daily individual AQI (IAQI) is calculated from the concentrations of individual pollutants, and the AQI value is determined to be the maximum IAQI of the six pollutants. When daily AQI is greater than 50, the pollutant that has the highest IAQI index is referred to as the daily ‘major pollutant’ contributing most to the air quality deterioration^8,24, 28^. When daily IAQI is greater than 100, air quality does not meet the CAAQS-Grade II level for 24-h average PM_2.5_, PM_10_, SO_2_, NO_2_, CO, or maximum 8-h average O_3_, and such days are considered ‘non-attainment days’^24^. The corresponding concentration limits of PM_2.5_, PM_10_, SO_2_, NO_2_, 24-h average CO, and O_3__8h when IAQI equals 50 or 100 are shown in Table 4.

$$AQI = \max \left\{ {IAQI_{1} ,IAQI_{2} ,IAQI_{3} , \ldots ,IAQI_{p} } \right\}$$

**Table 4:** Individual air quality index (IAQI) and corresponding pollutant concentration limit^52^.

IAQI	Pollutant concentration limit (μg m^−3^)									
	SO_2_		NO_2_		PM_10_	CO (mg m^−3^)		O_3_		PM_2.5_
	24-h average	1-h average^a^	24-h average	1-h average^a^	24-h average	24-h average	1-h average^a^	1-h average	8-h average	24-h average
0	0	0	0	0	0	0	0	0	0	0
50	50	150	40	100	50	2	5	160	100	35
100	150	500	80	200	150	4	10	200	160	75
150	475	650	180	700	250	14	35	300	215	115
200	800	800	280	1200	350	24	60	400	265	150
300	1600	^b^	565	2340	420	36	90	800	800	250
400	2100	^b^	750	3090	500	48	120	1000	^c^	350
500	2620	^b^	940	3840	600	60	150	1200	^c^	500

^a^1-h average concentration limits of SO_2_, NO_2_, and CO are only used in real-time reporting, and the 24-h average concentration limits of SO_2_, NO_2_, and CO are used in daily reporting.

^b^When 1-h average concentration limit of SO_2_ is higher than 800 μg m^−3^, the individual air quality index of SO_2_ is not reported and the reported individual air quality index of SO_2_ is calculated by 24-h average concentration limits.

^c^When 8-h average concentration limit of O_3_ is higher than 800 μg m^−3^, the individual air quality index of 8-h average concentration of SO_2_ is not reported and the reported individual air quality index of SO_2_ is calculated by 1-h average concentration limit.

where *IAQI is* individual air quality index and *p* is pollutant; and

$$IAQI_{p} = \frac{{IAQI_{Hi} - IAQI_{Lo} }}{{BP_{Hi} - BP_{Lo} }}\left( {C_{p} - BP_{Lo} } \right) + IAQI_{Lo}$$

where *IAQI*_*p*_ is individual air quality index of pollutant *p*, *C*_*p*_ is concentration of pollutant *p*, *BP*_*Hi*_ is high-value pollutant concentration limit when close to *C*_*p*_ (in Table 4), *BP*_*LO*_ is low-value pollutant concentration limit when close to *C*_*p*_ (in Table 4), *IAQI*_*Hi*_ is the individual air quality index corresponding to *BP*_*Hi*_, and *IAQI*_*LO*_ is the individual air quality index corresponding to *BP*_*LO*_.

**Figure 6:**
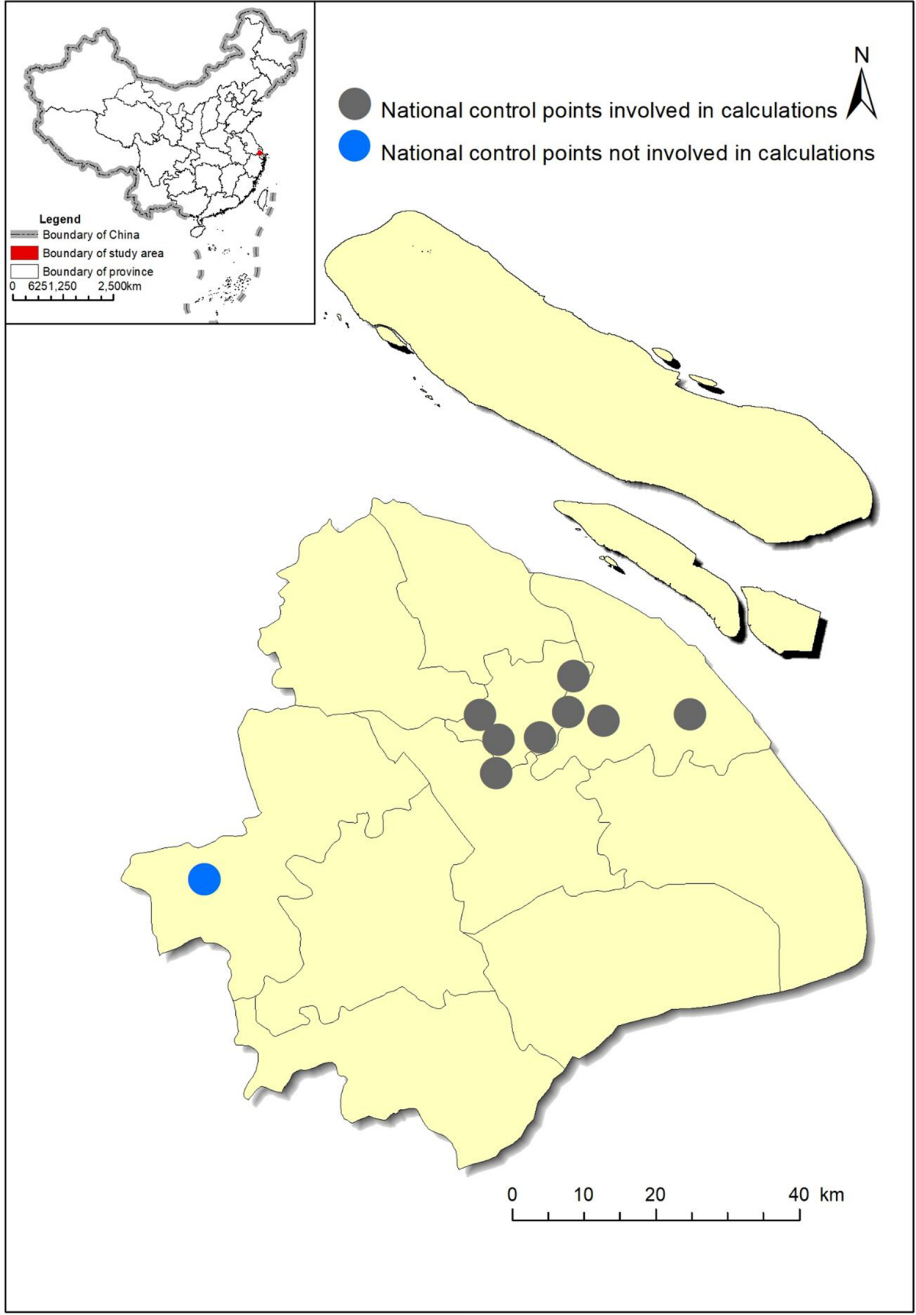
Location of national air quality monitoring stations in Shanghai municipality.

Data on the real-time daily average concentrations of PM_2.5_, PM_10_, CO, NO_2_, and SO_2_ and the maximum 8-h average concentration of O_3_ at nine national air quality monitoring stations (Fig. 6) were obtained from the Shanghai Environmental Monitoring Center. Data on different air quality levels were obtained from Shanghai Environmental Bulletin (2015–2017) and Shanghai Ecological Environmental Bulletin (2018), which is open-access (https://sthj.sh.gov.cn/hb/fa/cms/shhj/list_login.jsp?channelId=2144). Monthly meteorological data (*Prec*, *W*, *T*, and *RH*) from two ground-level monitoring sites were downloaded from the China Meteorological Data Sharing Service System (https://data.cma.cn/).

CO is measured using the non-dispersive infrared absorption method^8,51^, PM_2.5_ and PM_10_ are measured using the micro-oscillating balance method and the β absorption method^8,51^, and SO_2_, NO_2_, and O_3_ are measured by the fluorescence method, the chemiluminescence method, and the UV-spectrophotometry method, respectively^8,51^. Correlation analysis (using SPSS 16.0) was applied to determine the relevance of the six pollutants, meteorological factors, and socio-economic indicators. Independence and normality tests were performed before the correlation analysis. Pearson correlation analysis was performed when the data were normally distributed, otherwise Spearman correlation analysis was applied.

## Data Availability

All relevant data are available upon request from the authors.
